# The Practice of Power by Regional Managers in the Implementation of an Indigenous Peoples Health Policy in the Philippines

**DOI:** 10.34172/ijhpm.2020.246

**Published:** 2020-12-14

**Authors:** Ryan C. Guinaran, Erlinda B. Alupias, Lucy Gilson

**Affiliations:** ^1^Department of Extension Education, College of Agriculture, Benguet State University, Benguet, Philippines.; ^2^HPS Division, University of Cape Town, Cape Town, South Africa.; ^3^London School of Tropical Medicine and Hygiene, London, UK.

**Keywords:** Health Policy Implementation, Practice of Power, Indigenous Peoples, Philippines

## Abstract

**Background:** Indigenous peoples are among the most marginalized groups in society. In the Philippines, a new policy aimed at ensuring equity and culture-sensitivity of health services for this population was introduced. The study aimed to determine how subnational health managers exercised power and with what consequences for how implementation unfolded. Power is manifested in the perception, decision and action of health system actors. The study also delved into the sources of power that health managers drew on and their reasons for exercising power.

**Methods:** The study was a qualitative case study employing in-depth semi-structured interviews with 26 health managers from the case region and analysis of 15 relevant documents. Data from both sources were thematically analyzed following the framework method. In the analysis and interpretation of data on power, VeneKlasen and Miller’s categorization of the sources and expressions of power and Gilson, Schneider and Orgill’s categorization of the sources and reasons for exercising power were utilized.

**Results:** Key managers in the case region perceived the implementation of the new Indigenous health policy as limited and weakly integrated into health operations. The forms of power exercised by actors in key administrative interfaces were greatly influenced by organizational context and perceived weak leadership and their practices of power hindered policy implementation. However, some positive experiences showed that personal commitment and motivation rooted in one’s indigeneity enabled program managers to mobilize their discretionary power to support policy implementation.

**Conclusion:** The way power is exercised by policy actors at key interfaces influences the implementation and uptake of the Indigenous policy by the health system. Middle managers are strategic actors in translating central directions to operational action down to frontlines. Indigenous program managers are most likely to support an Indigenous health policy but personal and organizational factors can also override this inclination.

## Background

Providing healthcare that is responsive to the needs of the world’s nearly 400 million Indigenous peoples remains a challenge. Indigenous populations continue to have poorer health and social indicators than the general population.^1-4^ In the Philippines, Indigenous peoples comprise around 13% (10 to 15 million) of the total population^5^ and have worse health outcomes than the general population^6-9^ due to physical distance, poor quality of care and social and cultural exclusion.^5,10-12^

Recognizing these health inequities and in support of the country’s Indigenous Peoples Rights Act of 1997, the Department of Health (DOH), the National Commission on Indigenous Peoples (NCIP), and the Department of Interior and Local Government (DILG) issued a Joint Memorandum Circular (JMC) on Indigenous Peoples Health in April 2013. The DOH considered this a critical milestone and it was co-formulated by stakeholders from government, non-government, civil society and Indigenous peoples. The circular pertained to “Guidelines on the Delivery of Basic Health Services for Indigenous Cultural Communities/Indigenous Peoples” and was designed to improve healthcare in Indigenous areas by facilitating access, equity and culture-sensitivity in basic services.^5^

It is widely recognized that in low- and middle-income countries, new health policies commonly face implementation challenges.^13-15^ One challenge is the way in which implementing actors use their powerand these practices of power are acknowledged to be ‘at the heart of every policy process.’^16-18^ These compromise every individual’s perceptions, judgments and actions and they play out in decision-making processes and influence resource mobilization.^19^ As power is dynamic, it can be expressed in different forms such as domination, resistance or collaboration.^20^

Other challenges to implementation lie in the often overlooked dynamics of multilevel governance.^21^ A key issue is that as policies are cascaded across tiers, they are translated and reshaped by policy actors exercising their power – and this can cause a thinning down of the policy intent.^22^

Literature on power in implementation, especially in low- and middle-income countries is still limited.^16^ Furthermore, there has been limited investigation about the implementation of health policies for Indigenous rights and the influence of Indigenous managers, or these policies’ impacts.^23,24^ It is important to consider these health managers as they are important actors in Indigenous care systems^25^ and exercise discretionary power over a range of policy actors and processes (eg, service delivery, citizen engagement, the policy itself, and managerial processes).^26^

This study sought then to analyze the implementation of the 2013 JMC by DOH managers working at the regional level of the Philippines’ health system. Administratively, the country is organized into 17 regions which host local government units (LGUs) that include 81 provinces, and the 1490 municipalities and 145 cities that are geographically situated within these provinces.^27^ Although authority for the management and delivery of health services was devolved to these LGUs in 1991,^28^ the national DOH remains important in the health system. The DOH provides a range of resources to LGUs (additional funds, augmentation of personnel, capital outlay, insurance, health goods and services) and its regional offices also assist LGUs by providing technical assistance in health matters.^27,29^ As mid-level managers, regional DOH officials mediate centrally-led action and create spaces for locally- responsive decision-making.^30^

This study aimed specifically to track the major activities related to the implementation of the 2013 JMC on Indigenous Peoples Health in the case region and determine how the health managers exercised power and with what consequences for implementation processes. It also investigated the sources of power that the managers drew on and their reasons for exercising power. The paper, thus, employs actor-centric analysis and makes a contribution to health implementation literature at the subnational level and to Indigenous health policy implementation literature.

## Methods

### Design

The study was a qualitative case study that sought to provide an in-depth description of the ‘how and why’ of the Indigenous health policy’s implementation in a regional setting.^31^

### Participants

Participants were drawn from a purposefully chosen regional office, selected because it serves as one of the four (out of 17) regions in which Indigenous peoples comprise at least 40% of the population.^32^ Within this office, twenty-five health managers were purposively selected to be interviewed because they knew about the 2013 JMC (Indigenous health policy); in addition, one provincial health officer was identified for interview through the snowballing method. Of these 26 participants, 19 were women and 7 were men, and only four managers were non-indigenous. To ensure confidentiality, the regional office of focus is anonymized in the paper, and simply referred to as the case Regional Office.

### Data Collection

Data were gathered from one-on-one semi-structured in-depth interviews and document analysis. Data collection was primarily undertaken from September 2018 to February 2019 with follow-up interviews to validate insights from October 2019 to January 2020. The Director of the DOH Regional Office approved the study and authorized interviews with the staff and researchers’ access to regional documents. The Regional Office of the NCIP also approved the conduct of the study. The participants were primed about the nature and objectives of the research and informed consent was secured from all, using a form based on the World Health Organization (WHO) template. The interview guide probed their assessment of the implementation of the Indigenous health policy by the regional office, the key actors, and the facilitators and barriers to policy implementation. Each interview, which ran from 30 to 70 minutes, was audio-recorded and supplemented by note taking, with the researcher validating written key findings with the informant after the interview.

Organizational documents assist in reviewing the wider context that a case inhabits and their analysis requires proper identification, selection, appraisal and consequent synthesis of data.^33,34^ Fifteen documents at the DOH Regional Office were reviewed as these pertained to implementation activities related to the Indigenous policy. These were a national report on the conceptualization of the Indigenous health policy, minutes of four Regional Inter-Agency Committee meetings, seven evaluation reports on culture-sensitivity workshops done in the region, two related office circulars issued by the regional department, and an office performance commitment and review form.

### Data Analysis

The widely-used framework approach to thematic data analysis was applied to interviews and documentary textual data, requiring identification of the commonalities and relationships between sets of data and descriptive conclusions based on themes.^35^ Interviews were transcribed and the transcripts were read to ensure familiarization with the content. As transcripts were read line by line, codes were applied to content deemed as significant and the codes were sorted into categories that formed the analytical framework. The major events in policy implementation were inductively identified primarily from the documents, supported by data from the interviews. In examining the practice of power in implementation, the analysis and interpretation of data utilized a-priori themes^36^ drawn from two analytical frameworks. First, in considering the forms of power, VeneKlasen and Miller’s categorization of power as power over, power with, power to, and power within (Table 1) was used.^20^ Second, in considering how the micropractices of power play out in policy implementation specifically, we drew on Gilson and colleagues’ work to consider the ‘sources’ of power underpinning its exercise (the how question) and the ‘reasons’ why actors exercise power in such ways (triggers for exercising power).^26^ In this study, the specific subthemes of sources and reasons were inductively derived from the interview data.

**Table 1:** Four Expressions of Power

**Expressions of Power**	**Definition**
Power over	Having power involves taking it from someone else and then using it to dominate and prevent others from gaining it
Power with	Has to do with finding common ground among different interests and building collective strength
Power to	Refers to unique potential of every person to shape his or her life and world
Power within	Has to do with a person’s sense of self-worth and self-knowledge

Source: VeneKlasen and Miller.^20^

To reinforce the validity and reliability of the study, multiple sources of data were collected and analysed, established frameworks were used in the analysis (see below) and the preliminary analysis was presented to the participants who reviewed the draft report in a validation process.^37^

## Results

To understand the policy implementation dynamics of this case, the findings are presented in three sub-sections. First, a narrative of the major events in policy development and implementation across the country and in the region of focus is presented. Second, a more detailed exposition of the power practices that underpinned policy implementation in this region is presented, together with, third, an examination of the sources of power and the factors that triggered these practices of power.

### Overview of the Indigenous Health Policy Implementation Process

In considering the implementation process, we first consider “Policy operationalization at the national level”- largely informed by analysis of documents and second, the “Adoption and implementation at the Regional DOH”- which relied on interview data.

### Policy Operationalization at the National Level

**Figure 1:**
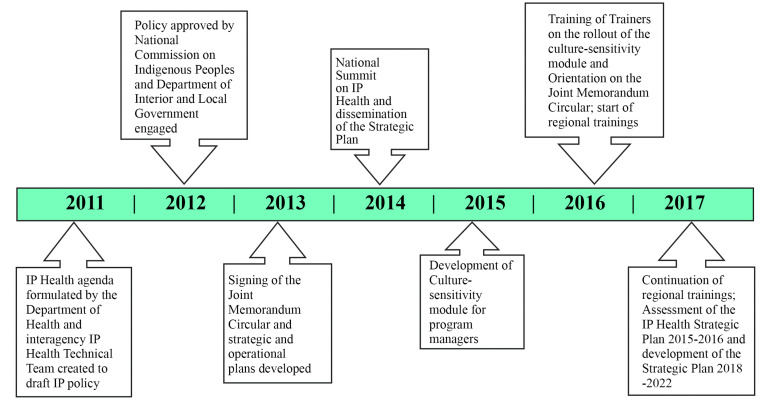
Timeline of the Indigenous Health Policy Formulation and Implementation, 2011-2018. Source: documentary materials.

Figure 1 outlines the timeline of policy development, from initiation by the national DOH in 2011. The 2011-2012 development of the draft policy by the DOH, the NCIP and the DILG involved the technical team consulting government agencies, Indigenous peoples’ organizations and LGUs. Adopted in 2013, the resulting policy recommended enhancement of general health guidelines to fit Indigenous community conditions and adopted a health systems approach (Box 1).^5^

**Box 1. Core Strategies of the DOH-NCIP-DILG JMC**

1. Health governance establishing mechanisms for leadership, accountability, stewardship and meaningful participation of Indigenous peoples in policy-making and decision-making;
2. Human resources for health addressing shortage in quantity and quality of human resources by increasing the number, improving capacity and providing other mechanisms that will manage such shortages (recruitment of Indigenous peoples in health workforce, scholarships, and CSTs for health workers);
3. Infrastructure and equipment addressing the shortage in quantity and quality of facilities and incorporating appropriate Indigenous designs/materials;
4. Timely supply and delivery of essential medicines and its alternatives ensured to all healthcare facilities and Indigenous communities;
5. Service standards serving as quality control mechanism for services rendered that will ensure access, adequacy and appropriateness for the Indigenous population served;
6. Financing sources and management identifying all possible sources of funds and resources for Indigenous health service delivery.

Abbreviations: DOH, Department of Health; NCIP, National Commission on Indigenous Peoples, DILG, Department of Interior and Local Government; CSTs, culture-sensitivity training; JMC, Joint Memorandum Circular.

In 2014, an operational and strategic plan was formulated at the national level and rolled out to the LGUs through the regional health offices, each of which designated an Indigenous Peoples’ health coordinator from among their existing personnel. The national DOH also prepared a training module for JMC orientation/culture-sensitivity and all regional Indigenous Peoples’ health coordinators were called to a training of trainers workshop in 2016. Thus, it was only after 2016 that policy implementation at the regional level began.

The 2014 strategic plan left the specific details of implementation to the DOH regional offices, stating that, initially, the implementation budget should be drawn from the recurrent budget of the offices, competing with other policy priorities. With the policy’s broad health system focus (Box 1), implementation at the regional level was, then, mainly dependent on the leadership of the regional Indigenous Peoples’ health coordinator to mobilize resources and the support of regional health office management to support its integration into other activities.

### Adoption and Implementation at the Regional DOH

The JMC stipulates that the DOH Regional Office provide technical assistance to the LGUs, conduct training and capacity-building, advocate to LGUs and monitor and evaluate activities covered by the policy. The regional DOH program managers who were interviewed judged that, after 2017, implementation of the Indigenous health policy in their region was, overall, “limited, superficial, weakly integrated” as very few relevant activities had been implemented. The main regional accomplishments from 2017 to 2018 were the JMC orientation and culture-sensitivity trainings (CSTs) and the conduct of the first regional Indigenous Peoples’ Health Summit.

The key managers involved in these key activities were the Indigenous Peoples’ health coordinator, the Planning Officer (coordinated internal systems for planning), the Local Health System Section (LHSS) Chief and Program Assistant (supported local health systems priorities), the regional office’s two Training Specialists (handled competency development), and the Licensing and Regulatory Officer (enforced regulatory policies). These managers were all part of the supervisory division overseeing operations in the DOH Regional Office. Other actors (program managers) were regional DOH coordinators of national public health and disease-specific programs (eg, Coordinator for the Traditional and Alternative Healthcare program) who belonged to the local health support division for the DOH’s vertical programs.

In practice, three different Indigenous Peoples’ health coordinators led the Indigenous health program across 2017-2018, given staff promotion and reassignment in the office. The third coordinator had just taken over when the study was conducted.

The first coordinator mobilized regional DOH employees and LGU point persons as participants in the CST workshops. However, because she was not indigenous and felt inexperienced in this area of work, she outsourced the substantive work of the training and played only an administrative role. The workshops included the development of action plans to support JMC implementation and during the first regional workshop (June 2017), 37 specific activities were identified. However, the Planning Officer noticed that the coordinator did not actively track and monitor the outputs of the workshop, and review of evaluation reports showed that after a year only nine projects/initiatives had been implemented.

As there were only a few participants in the first workshop, Training Specialist B, who is Indigenous, organized another CST workshop for regional program managers in December 2017. Although such training was not part of her planned activities for the year, she allocated funds and engaged regional managers to attend. The December workshop generated 81 specific activities to support policy implementation but again, no officer was monitoring compliance to the plans. A subsequent review of the plans again showed limited progress, although a few regional program managers were successful in infusing new approaches into their programs to make them more culture-sensitive.

The second Indigenous Peoples’ health coordinator showed her interest in the position during the LGU trainings when she shared ideas on Indigenous health based on her vast experience as an Indigenous nurse in primary healthcare. After the endorsement of her new role, however, her capacity to track regional program managers’ and provincial governments’ initiatives was limited by the absence of training output documents. She was also occasionally absent from work due to health reasons. Her immediate supervisor, the LHSS chief who is an Indigenous physician, and an LHSS program assistant, who is an Indigenous nurse, eagerly represented her in Indigenous health activities during her absences.

A major activity during the second coordinator’s stint was the first regional Indigenous health summit in which local studies and initiatives on Indigenous health were presented to regional, provincial and municipal stakeholders. The LHSS chief and the program assistant, the Planning Officer, and the Training Specialist A led this health summit and their alliance was able to influence the regional management committee to hold the summit. During the event, working committee members were present but the Indigenous program coordinator was inadvertently left out. The LHSS chief clarified, “I’m sorry that we missed out on her in the office order. The instruction from the management was to limit the regional DOH attendees to the technical working committees since we were expecting many external participants. We took over her role in much of the planning and implementation, that’s why.”

After the CSTs in the LGUs, the policy was put to test when one Provincial Health Board (an advisory board to local policy-making), sent a resolution to the regional DOH to put on hold a pilot vaccination program in their Indigenous locality, asking that the DOH seek their consent first. As a guiding principle in the Indigenous Peoples’ health policy was respect for Indigenous rights through participatory processes, the vaccination was put on hold and a formal response from the regional DOH office only came after some months.

### Digging Deeper: Practices of Power in Implementation of The Indigenous Health Policy

To better understand the dynamics of the implementation process described earlier, this section presents an analysis of health system actors’ exercise of power, drawing on interview data. It considers actor interactions at three main administrative interfaces within the health system: DOH National Office/DOH Regional Office; within units in the DOH Regional Office; and DOH Regional Office/the LGU.

For each interface (column 1), Table 2 identifies the key actors (column 2), their distinct obligations (column 3) and practices of power (column 4) which shaped the overall experience of the Indigenous health policy implementation. Although some practices supported implementation, the dominant experience was that the exercise of bureaucratic power over other actors acted to thwart and constrain effective implementation.

**Table 2:** Summary of Key Actors and Their Practices of Power Across the Interfaces

**Administrative Interface**	**Actors**	**Function of Actor**	**Practice of Power (Drawing on VeneKlasen and Miller** ^20^ **)**
DOH National Office and DOH Regional Office	DOH National Office	Develop national plans, set technical standards, and formulate guidelines on health	Power over DOH Regional Office and LGUs, through priority-setting, standard-setting, resources, performance-monitoring and targets in policies and programs
	Regional Licensing and Regulatory Officer	Assess health providers if they are in compliance with standards and regulatory policies provided by DOH National Office	Power within and power to act to accommodate Indigenous variations when monitoring facilities and services
Within units/managers at the DOH Regional Office	LHSS Chief; Program assistant; Training Specialist A; Planning Officer	Assess and support priorities in local health systems development Facilitate development of competencies of staff Coordinates sectoral and internal systems and processes for health planning and program development	Power within and power with as they formed an alliance to organize the regional Indigenous Peoples Health Summit
	Training Specialist B	Facilitate development of competencies of staff	Power to act in organizing another CST for regional office personnel (utilizing her unit’s budget) since previous training organized by Indigenous Peoples’ health coordinator was not well-attended
	Program managers/coordinators of vertical programs	Manage vertical health disease-specific and family health programs	Power to infuse Indigenous innovations in their programs Power to not infuse Indigenous innovations due to other priorities, and lack of cultural competence
	Indigenous Peoples’ health coordinators	Act as point person for the DOH Regional Office functions relative to the Indigenous policy	Power to drive adoption of relevant policy provisions by program managers in their tasks (but this practice of power was perceived as weak by regional managers)
DOH Regional Office and the LGU	DOH Regional Office	Provides technical assistance, training, capacity-building, and advocacy to LGUs regarding the health policy, monitors and evaluates	Power over LGUs through priority-setting, resources, performance monitoring and set targets in programs
	Indigenous Peoples’ health coordinator	Act as point person for the DOH Regional Office functions relative to the Indigenous health policy	Power over provincial LGUs to conduct JMC Orientation and CST
	Provincial Health Officer and Provincial Health Board members	Serve as an advisory committee to policy-making on health matters in the provincial LGU	Power with co-members and power to act in challenging a pilot program of the DOH Regional Office in their Indigenous locality

Abbreviations: DOH, Department of Health; LHSS, Local Health System Section; CST, culture-sensitivity training; LGU, local government unit; JMC, Joint Memorandum Circular.

### DOH National Office/DOH Regional Office Interface

The DOH national office exerted its power over the DOH regional office, through imposing national priorities, as well as through its inherent authority and resources (Table 2). In general, regional program managers’ behavior was strongly driven by central office requirements, especially performance targets set for each program. However, although the Indigenous health policy originated from the Central Office, it did not set Indigenous health indicators for these programs:

*“I think the first priority of program managers is what the Central Office asks from us. What are our targets per program? Indigenous health- it’s not it”*(Training Specialist B).

As a result, regional program managers often took Indigenous health lightly, especially because they had concerns about their workloads and saw the policy’s implementation as an added task. They also perceived that Indigenous Health was a separate vertical program, not their responsibility, since it had its own coordinator. Even those managers who were able to infuse culture-sensitivity activities in their programs were not able to sustain this focus over time. It “lies low” (as some participants articulated) as there was no institutionalized monitoring of such pro-Indigenous health initiatives.

The Central Office’s formal clout (power over) only worked well in cascading the JMC orientation/CST workshops to the Regional Office and the LGUs because these activities were prioritized in the national Indigenous Health policy strategic plan.

Nonetheless and despite the strong norm of top-down implementation (central to regional), the practice of discretionary power by some regional program managers who were deeply connected to their sense of indigeneity (power within) supported policy implementation. An example is the Licensing and Regulatory Officer (Table 2) who was Indigenous. Noting that the national standards do not explicitly articulate adjustments based on Indigenous culture or conditions, she exercised her power to accommodate Indigenous initiatives:

*“Criteria are set from above (Central). This can hinder how you inject culture-sensitivity in what you do. Occasionally, we do not strictly follow some guidelines if there are Indigenous alternatives so long as the patients’ safety is not compromised. For example, I allow the ordinary bed preferred by Indigenous Peoples (over the delivery bed with stirrup) in the birthing centers”*(Licensing and Regulatory Officer).

### Interface Among Units/Managers in the DOH Regional Office

At the regional office, meanwhile, contestation and collaboration surfaced between peers (Table 2). The majority of program managers were not able to accomplish the Indigenous health innovations that they planned during their CST workshops. They stated that they prioritized their own program deliverables and lacked cultural competence skills. However, with information and skills acquired from the workshops, a few program managers utilized their power to redesign their projects to be more culturally sensitive and provided resources to implement them. For example, the Coordinator of the Traditional and Alternative Healthcare program (a vertical program set by the National DOH) incorporated questions on Indigenous cultural practices in her monitoring tool.

*“In our provincial training on reproductive health, we adopted the tool on assessing cultural practices that we learned during the training. We had a good output”*(Development Management Officer).

The program managers’ power to instill culture-sensitivity reinforced policy implementation but the lack of institutionalized monitoring made sustaining changes difficult.

*“The assumption is that since you were oriented on the JMC and culture-sensitivity then you should apply it already in the program that you handle. But there’s no monitoring process or follow up”*(LHSS Chief).

In addition, weak supervision by the Indigenous Peoples’ health coordinators was felt to deter policy implementation. The first coordinator was criticized as merely complying with national directives and not initiating regional innovations by other managers who wanted more support for Indigenous health.

*“I think she found the program challenging. She would say she is not Indigenous. Maybe she could not relate or appreciate it”*(Planning Officer).

*“What the coordinator did was more of downloading. But we wanted attention to Indigenous Health. For example, culture-sensitivity trainings were done but the coordinator did not collect provincial output. That’s a missed opportunity to advance the program”*(LHSS Chief).

Other regional actors then also took action to support policy implementation. For example, Training Specialist B exercised her power to organize a second JMC orientation/CST workshop using funds from her office, having noted that the first workshop was not well-attended. Another example of “taking over” tasks formally allocated to the Indigenous Peoples’ health coordinator was in relation to the first regional Indigenous Peoples’ Health summit, when the LHSS chief and a program assistant became involved in the organizing and planning committee. An unexpected alliance among the LHSS chief, program assistant, Training Specialist A, and the Planning Officer emerged to provide a strong support for the health summit, with the shared motivation of being Indigenous. Their work mandates (support, planning, training) allowed them to influence other program managers. *“With the weakness of the designated Indigenous Peoples’ Health managers, some of us have to step in (to advance the program),”* the Planning Officer added.

### DOH Regional Office/LGU interface

The practice of authoritative power by the regional office towards the LGUs (Table 2), or the top-down approach (power over) worked in two different ways. It helped the Indigenous health coordinators roll out the JMC orientation/CST workshops in all LGUs. With their authority as the regional Indigenous Peoples’ Health coordinator, they mobilized LGUs to gather stakeholders as participants to the workshops. However, this authoritative power also became a hindrance to policy implementation when regional program managers continued to use generic designs and approaches (deficient in indicators on Indigenous Peoples and not localized for the LGU setting) crafted by the national office.

*“The implementation practice is top-bottom, with limited initiatives for consultation, and limited or no localization of interventions”*(Program Manager Maternal and Child Health).

*“DOH is too rigid and strictly follows manuals of operations which have little consideration for Indigenous culture or local contexts”*(Provincial Health Officer).

The norm in this interface is that the top-down health programs are accepted, not resisted, by the LGUs. This tradition was challenged, though, when a provincial LGU through its Provincial Health Board asked that the DOH seek their consent before implementing a DOH pilot vaccination program in their Indigenous locality, putting the program on hold for several months. The regional office lacked specific guidelines for cultural negotiation with stakeholders but, as they became more aware of Indigenous Peoples’ health policy, the LGUs increasingly expected that the regional office should be proactive in ensuring culture-sensitivity in programs and processes.

*“For all new programs in the future, please get our consent-that’s our right. We have laws like Indigenous Peoples Rights Act so we can refine programs”*(Provincial Health Officer).

*“We don’t have specific guidelines on the process of cultural negotiation with LGUs”*(Planning Officer).

### Explanations: Sources and Reasons for the Practice of Power

This section, finally, explains how and why actors exercised power to undermine or support the Indigenous health policy’s implementation by drawing from interview data in considering the personal and organizational factors that acted as sources of power (Table 3) and as triggers (Table 4) for the exercise of power.

**Table 3:** Personal Factors as Sources or Reasons for Practice of Power by the Regional Office Managers^a^

	**Supporting Implementation**	**Constraining Implementation**
Sources underpinning the exercise of power (the how question)	Value judgment *“It is the right thing to do”*(Program Assistant). *“We accommodate as long as safety is not compromised; standard of care is not sacrificed”*(Regulation and Licensing Officer). Intrinsic motivation linked to indigeneity *“I am Indigenous. That’s my driving force”*(Program Assistant). *“We have Indigenous wisdom and technology that could be integrated in health programs”*(Regulation and Licensing Officer). Knowledge and skills relevant to tasks (cultural competence) from training, education, experience Planning Officer: social science educational background, previous work in Indigenous areas; Training Specialist A: IP Focal Person in previous work; LHSS Chief: previous medical work in Indigenous area; Program Assistant: previous work in Indigenous development field; Regulation and Licensing Officer: previous employment in the NCIP.	An attitude of low regard for what is Indigenous *“Why did we imbibe Western culture so easily? Because of our colonial history. It degraded the Indigenous peoples’ spirit. ‘IP’ is associated with ‘superstition, backward, substandard.’ They got our psyche. Nobody wants to be seen as backward”*(Provincial Health Officer). *“There is doubt in ‘traditional medicine’ compared to the scientific data written in our books”*(Training Specialist B). Lack of knowledge and skills on Indigenous health and culture/cultural competence Lack of awareness on Indigenous culture and issues *“We lack awareness on Indigenous peoples’ issues”*(Planning Officer).
Reasons why power was exercised in these ways (the triggers for exercising power)	Alignment of personal values with policy *“I’m proud to be IP so my principle is you cannot say you are IP if it doesn’t show in your actions and work”*(Training Specialist A). Personal commitment and motivation *“I look for that (culture-sensitivity) because I’m Indigenous”*(Licensing Officer).	Lack of commitment Unclear concept or limited understanding of what Indigenous health is *“On Indigenous health, if your assumption is that because we are all Indigenous, that’s just the coverage of your services. It doesn’t tell about the quality or culture-sensitivity. We lack a deeper understanding of what culture sensitive means for our Indigenous peoples’ services”*(LHSS Chief). Unclear about the need for ‘Indigenous Peoples health’ in the region * “Our weak regard for our Indigenous health locally may be unintentional because we are the majority here in our region. Maybe if we are the minority, we would be more conscious of it”*(Planning Officer). Limited skills for local Indigenous Peoples health *“In our transcultural nursing, we learned about other countries’ culture like Mexico’s but not ours. We were groomed for the NCLEX”*(Training Specialist B). *“There’s no Indigenous Peoples health in our educational curriculum”*(Planning Officer). *Lack of guidance and incentives* Indigenous Peoples Health is seen as an additional task/workload *“Everybody’s overloaded with many programs. Personally I want to see the integration of Indigenous health in the programs. But I see the workload as a barrier”*(Training Specialist). Indigenous health activities in Individual performance target not institutionalized and sustained. Policy not clear about instances when Indigenous culture may be in conflict with DOH standards/other policies.

Abbreviations: DOH, Department of Health; LHSS, Local Health System Section; NCIP, National Commission on Indigenous Peoples, NCLEX nursing examination to practice in USA. ^a^Categories adapted from Gilson et al.^26^

**Table 4:** Organizational Factors as Sources or Reasons for Practice of Power by the Regional Office Managers^a^

	**Supporting Implementation**	**Constraining Implementation**
Sources underpinning the exercise of power (the how question)	Policy itself - Provided authority moreso as a JMC of three agencies, direction and specific roles of stakeholders Organizational and management environment - Coordinator identified with clear responsibilities Resources available - For capacity-building from Indigenous Peoples health unit and other managers	Weak lines of accountability *“We have to review processes and forms to capture Indigenous peoples’ concerns and action”*(Planning Officer). Many/competing organizational priorities
Reasons why power was exercised in these ways (the triggers for exercising power)	Organizational relationships Managers form working relations to support IP health activities apart from their usual tasks	Management processes that are: Top-down Lacking processes on cultural negotiation Lacking efficiency in management succession- Frequent changes in program leadership *“Indigenous peoples’ health policy success in the regional office is also dependent on the coordinator who would continuously advocate it. (Indigenous Peoples Health coordinator changed thrice in a span of two years)”*(Planning Officer).

Abbreviation: JMC, Joint Memorandum Circular. ^a^Categories adapted from Gilson et al.^26^

### Exercising Power to Undermine Policy Implementation

The majority of regional program managers exercised power in ways that constrained implementation because of personal factors such as having an attitude of low regard or inferiority for what is Indigenous (compared to mainstream, or ‘Western’ health concepts), and a lack of knowledge and skills on Indigenous health and cultural competence (Table 3, column 3). They had limited understanding of what Indigenous health is, were unclear about the need for Indigenous Peoples’ health in the region, and lacked cultural competence skills to implement local health innovations. A lack of guidance and incentives triggered weak support for the policy. Indigenous Peoples’ Health was seen as an additional task and defined health activities for Indigenous Peoples were not institutionalized in individual performance targets. The policy was also not clear about how to manage instances when Indigenous culture might be in conflict with DOH standards or other policies. At an organizational level, implementation was undermined (Table 4, column 3) by weak lines of accountability and competing organizational priorities. This was due to strong top-down management processes that did not emphasize Indigenous policy integration, the lack of policy and management procedures for cultural negotiation with LGUs, and the frequent changes in program leadership. Taken together, these personal and organizational factors supported practices of power or inaction that deterred the overall Indigenous health policy implementation in the region.

### Exercising Power to Support Policy Implementation

Some regional actors’ practices of power shaped initiatives that supported the policy implementation. Noticeably, the actors highlighted in Table 2 who exercised power within, power with and power to, to support implementation drew on personal sources of power (Table 3, column 2). These included value judgments (that the policy is beneficial, just and relevant), an intrinsic drive linked to indigeneity, and having the knowledge and skills relevant to implementation as a result of training, education, and experience. The triggers for their exercise of power were the alignment of their personal values with the policy and a personal commitment and motivation to contribute to the policy’s successful implementation.

The organizational (Table 4, column 2) sources of power drawn on to support implementation were: the policy itself, which clarified direction and the specific roles of stakeholders and had legitimacy because it was developed by three government agencies; the designation of a coordinator with clear tasks; and availability of resources for implementation. Supportive managers also formed alliances to support Indigenous health activities as shown in the expression of ‘power with’ among the Planning Officer, LHSS Chief, Training Specialist A, and the Program Assistant.

## Discussion

**Figure 2:**
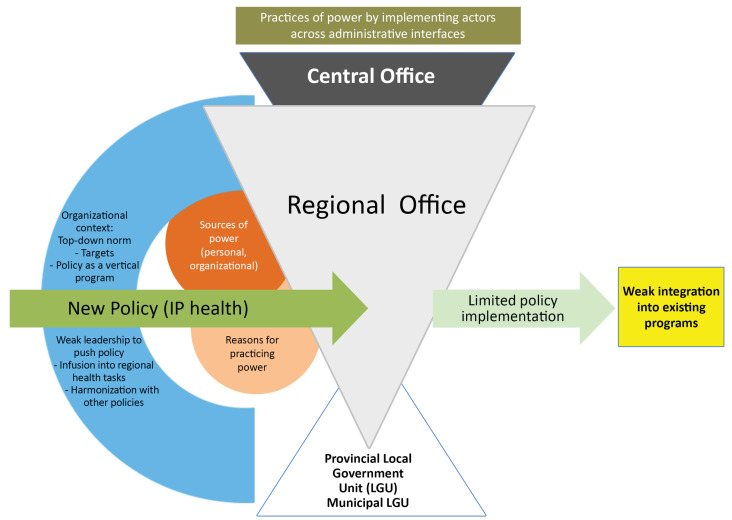
How Practices of Power Influenced the Indigenous Health Policy Implementation (Adapted from Gilson et al^26^).

This study aimed to examine the critical role of middle managers in health policy implementation in the Philippines. Specifically we focused on the subnational (regional) DOH office that provides leadership in health and functions as an arm of the national DOH in the region.^38-39^ This is the tier that directly interacts with LGUs in the devolved health system. Summarized in Figure 2, findings demonstrate that dynamic micro-practices of power at interfaces across system tiers were important in shaping the weak integration of the Indigenous health into existing programs, influenced both by personal and broader organizational factors.^22,40^

This study adds to the currently limited body of empirical literature that specifically examines the practice of power in implementation processes^16^ and, in particular, contributes insights about the implementation of indigenous health policies.

It demonstrates, first, how the top-down norm (power over) of the bureaucracy influenced implementation. In the Philippine health system, implementation was seen primarily as an administrative and hierarchical process cascaded from national level to operatives at the lower tiers as also observed in devolved arrangements in other countries.^41^ In this vertical process, centrally-set standards were established for specific programs, alongside performance targets which are powerful drivers of behavior when monitored.^42^ These led regional program managers to internalize the imposed authority and accountability,^43^ and work towards centrally-set goals. The top-down norm bestows on the DOH central office a ‘considerable power to shape the development of health services’ at lower levels such that what it prioritizes influences the priorities of the subnational and LGU levels.^44^ However, running counter to these bureaucratic norms, implementation of the Indigenous health policy was intended to be mainstreamed across programs and no program targets were set or monitored. As a result, regional program managers focused on their primary deliverables and regarded the Indigenous health policy as an added task that could be ignored. The dependencies across levels within the health system, meanwhile, meant that the weak prioritization of the Indigenous policy at the regional DOH office translated into a limited push at the LGU level.

Second, the study demonstrates the importance of mid-level managers in navigating implementation of the new Indigenous health policy. Leadership, the facility to rally to vision, inspire and provide momentum in a group of people,^45^ was vital for the rollout of the new Indigenous health policy. This policy had to be infused into the governance and operations of the health system and harmonized with other existing DOH guidelines before cascading it to the LGUs. These tasks depended on navigation by the Indigenous Peoples’ health coordinator, who possessed legitimate power based on position.^46^ Also, the coordinator was expected to lead successful policy implementation by exerting influence at all interfaces – upwards, laterally and downwards.^47^ It is more widely recognized that in addition to administering and coordinating programs and activities, mid-level managers offer leadership in engaging stakeholders.^48^ In addition, in implementing the indigenous health policy, a necessary leadership skill for mid-level managers is cultural negotiation as the norms of mainstream health services commonly conflict with Indigenous communities’ beliefs and practices.^49^ Instead of dismissing cultural conflicts in this policy’s implementation, mid-level managers need to work through its complexities.^50^

Third, the experience shows the ways in which the exercise of discretionary power can resist or support policy implementation. This policy’s implementation by program managers was constrained both by their lack of knowledge and skills on Indigenous health – a competency important in tailoring health programs to Indigenous contexts^51^- and their attitude of low regard for what is Indigenous – an issue revealed in other studies on implementing Indigenous cultural initiatives.^24^ These influences also suggest that the broader structural factors of contemporary educational and health systems may have negatively influenced the expressions of power in support of the Indigenous policy. Yet, at the same time, other regional managers, those who were themselves indigenous, used their discretionary power to support policy implementation. The use of discretionary power is a way of coping with challenging situations in bureaucracies.^17^ These managers drew power from the intrinsic motivation they derived from their own indigeneity, which was, moreover, triggered by the alignment of these personal values with the policy itself. In exercising this power, they also formed alliances with each other and tapped into an organizational feature, the ‘horizontal’ functions (training, assistance, planning, health support) for which they were responsible, to intrude into other programs. If they had been managing vertical health programs, they would likely have been constrained by the focus on public health program deliverables and the vertical silo mentality resulting from organizational and functional divisions.^52^

The links of indigeneity to policy implementation have also been shown more widely. For example, evidence suggests that policies meant for Indigenous communities strengthen Indigenous identity.^24^ In addition, it has also been recognized that employing Indigenous staff and leader-managers in organizations serving Indigenous populations helps to embed community’s cultural values in service delivery, increase Indigenous healthcare engagement, and facilitate cultural mentoring to non-Indigenous staff.^49,51,53,54^ However, this study noted that some Indigenous managers were not aware of their Indigenous culture and were less supportive of policy implementation. The mere presence of Indigenous managers in an organization does not, therefore, necessarily facilitate Indigenous policy implementation. Health managers, including those who are Indigenous, should be trained on cultural competence.^51^

## Conclusion

The practices of power by actors at key administrative interfaces are important influences over the implementation and uptake of the Philippines’ Indigenous health policy by the health system. This in-depth analysis of these practices reveals the power sources and triggers that provide the personal and organizational context for the manifestation of power. Subnational health managers or middle managers are strategic actors in translating central policies and directions to operational action down to frontlines. As such, probing into the use of their discretionary power is essential in health policy implementation analysis. Indigenous program managers are most likely to support an Indigenous health policy but personal and organizational factors can also override this inclination.

## Ethical issues

The university does not have an accredited Ethics Review Board. As a practice, the student’s advisory panel looks into and ensures that the researcher does ethical procedures. The student researcher observed the following steps:

1. Appropriate letters to the agencies/offices (Letters were sent to the DOH regional office and individual health managers for their acquiescence and approval to participate in the study; the NCIP in the region also approved the conduct of study and did not require a Free and Prior Informed Consent specific for indigenous communities).
2. Research participants were provided an informed consent form which they signed. The informed consent was patterned after the WHO template for informed consent in qualitative studies accessed from https://www.who.int/ethics/review-committee/informed_consent/en/.
3. To conceal identities of the managers, the region under study was referred to as simply the “case region” in the paper. No identifier such as name of province or city within its jurisdiction was written.
4. Validation of data gathered was done after each interview and key actors were provided drafts of the manuscript for their review.

## Competing interests

Authors declare that they have no competing interests.

## Authors’ contributions

All authors contributed equally to the development and finalization of the study protocol. RCG conducted the collection and analysis of data to produce the initial draft of the manuscript. All authors contributed to the review and editing of subsequent drafts to come up with the final manuscript.

## Authors’ affiliations

^1^Department of Extension Education, College of Agriculture, Benguet State University, Benguet, Philippines. ^2^HPS Division, University of Cape Town, Cape Town, South Africa. ^3^London School of Tropical Medicine and Hygiene, London, UK.

## Funding

This paper was supported by the Health Policy Analysis Fellowship Program of the Alliance for Health Systems and Policy Research and its mentorship team: Lucy Gilson, Maylene Shung-King, Irene Agyepong, and Jeremy Shiffman.

## Key Messages

**Implications for policy makers**

- Policy-makers must understand the role of subnational managers in the policy process as these middle managers are important actors in facilitating centrally-led policies and initiatives to local health system implementers. The policies do not just “pass through” them but are subjected to their interpretation and actions which directly affect policy implementation.
- Organizational context greatly influences the practices of power of policy implementers. Within an implementing organization, the policy should be clear to and understood by all and harmonized with other guidelines and policies before it gets disseminated. Strong leadership and institution-wide strategies are valuable in policy implementation especially for newly-introduced policies.
- Culture-sensitivity/cultural competence trainings are recommended for both Indigenous and non-indigenous health managers, healthcare providers and support groups providing services to Indigenous populations.

**Implications for public**

The public is not just at the receiving end of a health policy but is an active player in its implementation especially in providing feedback about the policy’s envisioned goals. The study found out how practices of power at different levels of a health system can negatively affect the implementation of an Indigenous health policy by delaying or undermining the implementation process. The public aspires for effective implementation and can actually influence these practices of power of policy implementers. Thus, as stakeholders in a policy aimed at health equity and access, intended beneficiaries have to be aware of the policy, be given the opportunity to be involved in its implementation, be a partner in policy monitoring and evaluation, and assert the provisions relevant to their issues. Indigenous peoples have a role in educating the health system about their culture and practices towards more culture-sensitive healthcare and better health outcomes.
